# *ESR1* mutations in metastatic lobular breast cancer patients

**DOI:** 10.1038/s41523-019-0104-z

**Published:** 2019-02-22

**Authors:** Christine Desmedt, Julien Pingitore, Françoise Rothé, Caterina Marchio, Florian Clatot, Ghizlane Rouas, François Richard, François Bertucci, Odette Mariani, Christine Galant, Charlotte Fribbens, Ben O’Leary, Gert van den Eynden, Roberto Salgado, Nicholas C. Turner, Martine Piccart, Anne Vincent-Salomon, Giancarlo Pruneri, Denis Larsimont, Christos Sotiriou

**Affiliations:** 1 Breast Cancer Translational Research Laboratory, Institut Jules Bordet, U-CRC, Université Libre de Bruxelles, 1000 Brussels, Belgium; 2 Laboratory for Translational Breast Cancer Research, KU Leuven, Herestraat 49, 3000 Leuven, Belgium; 3 Department of Pathology, Institut Curie, Paris Sciences Lettres Research University, 26 Rue d’Ulm, 75248 Paris Cédex05, France; 4 Department of Medical Sciences, FPO-IRCCS Candiolo Cancer Institute, University of Turin, SP 142 Km3.95, 10060 Candiolo, Italy; 5 Department of Medical Oncology, Centre Henri-Becquerel, CS11516 rue d’Amiens, 76000 Rouen, France; 6 IRON/Inserm U1245, Rouen University Hospital, 22, boulevard Gambetta, 76183 Rouen, France; 7 Predictive Oncology Laboratory, Institut Paoli-Calmettes, Centre de Recherche en Cancérologie de Marseille, Marseille, France; 8 Department of Pathology, Cliniques Universitaires Saint Luc, Brussels, Belgium; 9 Breast Unit, Royal Marsden Hospital, Fulham Road, London, SW3 6JJ UK; 10 The Breast Cancer Now Research Centre, The Institute of Cancer Research, London, SW3 6JB UK; 11 Department of Pathology, Sint Augustinus, Wilrijk, Belgium; 12 Institut Jules Bordet, Boulevard de Waterloo 121, 1000 Brussels, Belgium; 13 Division of Pathology, European Institute of Oncology, University of Milan, Milan, Italy; 14 Division of Pathology, Fondazione IRCCS Istituto Nazionale dei Tumori, via Venezian 1, 20133 Milan, Italy; 15 School of Medicine, University of Milan, via Festa del Perdono 7, 20122 Milano Milan, Italy; 16 Department of Pathology, Institut Jules Bordet, Boulevard de Waterloo 121, 1000 Brussels, Belgium

## Abstract

Invasive lobular breast cancer (ILC) represents the second most common histology of breast cancer after invasive ductal breast cancer (IDC), accounts for up to 15% of all invasive cases and generally express the estrogen receptor (ER, coded by the *ESR1* gene). *ESR1* mutations have been associated with resistance to endocrine therapy, however these have not been specifically evaluated in ILC. We assessed the frequency of *ESR1* mutations by droplet digital PCR in a retrospective multi-centric series of matched primary tumor and recurrence samples (*n* = 279) from 80 metastatic ER-positive ILC patients. We further compared *ESR1* mutations between IDC and ILC patients in metastatic samples from MSKCC-IMPACT (*n* = 595 IDC and 116 ILC) and in ctDNA from the SoFEA and PALOMA-3 trials (*n* = 416 IDC and 76 ILC). In the retrospective series, the metastases from seven patients (9%) harbored *ESR1* mutations, which were absent from the interrogated primary samples. Five patients (6%) had a mutation in the primary tumor or axillary metastasis, which could not be detected in the matched distant metastasis. In the MSKCC-IMPACT cohort, as well as in the SoFEA and PALOMA-3 trials, there were no differences in prevalence and distribution of the mutations between IDC and ILC, with D538G being the most frequent mutation in both histological subtypes. To conclude, no patient had an identical *ESR1* mutation in the early and metastatic disease in the retrospective ILC series. In the external series, there was no difference in terms of prevalence and type of ESR1 mutations between ILC and IDC.

## Genetics: Estrogen receptor mutations differ in early and metastatic tumors

Among a cohort of 80 women with metastatic lobular breast cancer, no patient had an identical mutation in the *ESR1* gene in both metastases and localized tumors. *ESR1* encodes the estrogen receptor targeted by endocrine therapy — thus, tumors harboring mutations in this gene may require different treatment strategies. Christine Desmedt  from KU Leuven, Belgium, and colleagues found that 9% of patients had metastases with *ESR1* mutations that were absent in primary breast tumors; another 6% had mutations in the primary tumors or in cancer-invaded lymph nodes that were not detected in distant metastatic sites. Comparisons with other datasets showed that the prevalence and distribution of *ESR1* mutations were not significantly different among women with lobular and ductal breast cancer, the two most common subtypes of the disease.

## Introduction

Invasive lobular breast carcinomas (ILC) account for up to 15% of all invasive breast cancer (BC) cases and represents the second most frequent histological subtype after invasive ductal BC (IDC), the latter also being formally referred to as invasive breast carcinoma of no special type.^1^ ILCs typically express the estrogen receptor (ER, coded by the *ESR1* gene) and lack *HER2* amplification. These tumors further differ from IDC in terms of clinical presentation, disease progression, and response to treatment.^2^ Recently, several studies on primary ILC have demonstrated that these two subtypes also present differences in terms of genomic, gene expression, protein, and immune features.^3–8^

Given the fact that ILCs are nearly exclusively ER-positive, those patients are almost always treated with endocrine therapy. While the majority of the patients do benefit from these treatments, a large proportion will present with *de novo* or acquired resistance. Although several mechanisms of endocrine resistance have been proposed,^9,10^ one of those that has been receiving more attention during the last years thanks to the increasing sequencing initiatives of metastatic breast tumors is represented by the recurrent mutations in the *ESR1* gene (reviewed by Jeselsohn et al.^11^).

ER is a member of the nuclear receptor superfamily and acts as a ligand-dependent transcription factor. Upon binding of estrogen, ER dimerizes and the α-helix of helix 12 is stabilized into an active conformation, allowing the binding of co-activators. This results in the binding of ER to several DNA sites to regulate the transcription of a multitude of genes involved in several physiological and cancer-related processes. The majority of the *ESR1* mutations are concentrated on two amino acids (Y537, D538) in the ligand-binding domain. These mutations have been reported to lead to ligand-independent activation.^12^

Mutations affecting the *ESR1* gene have been detected mainly in metastases from ER-positive/HER2-negative breast tumors, at a frequency ranging from 5 to 25%, when considering series with >20 interrogated metastases.^5,13–18^ The largest cohort of BC metastases with information about the *ESR1* mutational status is the one derived from the prospective clinical sequencing initiative from the Memorial Sloan Kettering Cancer Center (MSKCC-IMPACT^18^), which reported mutations in 107/835 (12.5%) metastatic BC patients (source: cbioportal.org^19,20^). Besides the detection in metastatic tissue, several studies have also interrogated the presence of these mutations in circulating tumor DNA (ctDNA) in institutional cohorts^15,21,22^ or in the context of clinical trials.^23–25^ The prevalence of *ESR1* mutations was much higher here, ranging between 14.8 and 31.5% in the institutional series and between 25.3 and 39.1% in the trials. Results are consistent across the cohorts in reporting higher *ESR1* mutation rates in patients treated with aromatase inhibitors in the metastatic setting.^15,22^

So far the prevalence of these mutations has been extremely low in primary tumors, with 0.5% mutated samples detected by next-generation sequencing (NGS) in the large cohort from The Cancer Genome Atlas (TCGA, source: cbioportal.org^3,19,20^). Recently, two studies using the more sensitive droplet digital PCR (ddPCR) reported higher frequencies in primary tumors of 2.6 and 7%, respectively.^21,26^ Two series of primary cancers from patients who recurred revealed also increased rates of 3 and 3.5% using NGS.^13,18^ Of interest, the mutant allele frequencies were generally very low in these primary tumors, rarely above 1%, suggesting that these mutated cells likely represent a minor subclone.

While the increased prevalence in the metastatic setting suggests that these mutations are acquired, few studies have extensively compared matched primary and metastatic samples to exclude that the *ESR1* mutations detected in the metastatic disease were actually already present in a rare subclone of the primary tumor.^13,16,17^ So far, the comparison on the highest number of matched primary and metastatic ER-positive samples was reported by Fumagalli et al.^16^ using a mutational hotspot qPCR assay on 37 patients, and none of the 4 *ESR1* mutations present in the metastatic samples were detected in the matched primary. However, given the higher rates of *ESR1* mutations detected with ddPCR, we hypothesized that deeper sequencing methods could maybe identify additional matched mutations. If the *ESR1* mutations were pre-existing in the primary tumor and led to endocrine resistance, this would have important therapeutic and monitoring implications.

Since tumors harboring *ESR1* mutations may require a different endocrine treatment strategy,^27^ it is of utmost importance to identify these. However, no study has ever specifically evaluated the presence and distribution of *ESR1* mutations in metastatic ILC. In this study, we therefore assembled a retrospective multi-centric cohort of matched normal, primary and metastatic samples from metastatic ER-positive ILC patients and evaluated the most frequent *ESR1* mutations using ddPCR. To further compare the frequency and distribution of the mutations between IDC and ILC patients, we interrogated the metastases from MSKCC-IMPACT^18^ and ctDNA from SoFEA and PALOMA-3.^24^

## Results

### Characteristics of the retrospective metastatic ILC cohort

We retrospectively identified 129 metastatic ER-positive ILC patients from 6 European institutions fulfilling the initial eligibility criteria. From these patients, following block retrieval and central pathology review, 94 were eligible. Sufficient DNA was available from the primary, the metastatic as well as normal tissue samples for 80 patients. Whenever possible, we also collected samples from involved axillary lymph nodes (Supplementary Figure 1). The patients and samples (*n* = 279) characteristics are listed in Table 1 and Supplementary Table 1. In brief, 71% of the patients were older than 50 years, 18% were de novo metastatic, 73% had primary tumors larger than 2 cm, and 65% had axillary lymph nodes involved at the time of diagnosis. The majority of the tumors were HER2-negative (84%) and grade 2 (61%). Of interest, the metastases from six patients lost ER expression in their metastatic sample. The most represented primary ILC histological subtypes were classic (56%); mixed non-classic (21%), and trabecular (16%). The origin of the analyzed metastases is consistent with the metastatic patterns observed for ILC patients,^28^ with bone metastases being the most frequent (24%), followed by metastases from reproductive organs and the skin (both 14%), and metastases from the gastro-intestinal tract and peritoneum (both 8%).

**Table 1:** Patient and sample characteristics of the retrospective metastatic ILC cohort

	*N* (%)
Age at primary diagnosis	
Median (min–max)	55 (33–81)
<50	23 (28.8)
≥50	57 (71.3)
De novo metastatic	
No	66 (82.5)
Yes	14 (17.5)
Histological primary tumor size	
<2 cm	20 (25.0)
≥2 cm	58 (72.5)
Unknown	2 (2.5)
Histological nodal status	
Negative	27 (33.8)
Positive	52 (65.0)
Unknown	1 (1.3)
Histological grade primary	
1	15 (18.8)
2	49 (61.3)
3	15 (18.8)
Unknown	1 (1.3)
PgR status primary	
Negative	11 (13.8)
Positive	66 (82.5)
Unknown	3 (3.8)
HER2 status primary	
Negative	67 (83.8)
Positive	6 (7.5)
Unknown	7 (8.8)
Ki67 primary	
<20%	38 (47.5)
≥20%	25 (31.3)
Unknown	17 (21.3)
ER status metastasis	
Negative	6 (7.5)
Positive	66 (82.5)
Unknown	8 (10.0)
Main histological subtype primary	
Alveolar	1 (1.3)
Classic	45 (56.3)
Mixed non-classic (incl. pleomorphic)	17 (21.3)
Solid	3 (3.8)
Trabecular	13 (16.3)
Unknown	1 (1.3)
(Neo)Adjuvant chemotherapy	
Yes	64 (80.0)
No	16 (20.0)
Organ metastatic sample^a^	
Bone	20 (23.5)
GI tract	7 (8.2)
Local relapse	3 (3.5)
Liver	8 (9.4)
Lung	8 (9.4)
Lymph nodes	6 (7.1)
Peritoneum	7 (8.2)
Reproductive organs	13 (15.3)
Skin	13 (15.3)
Timing metastatic sample	
At first diagnosis metastatic disease^b^	62 (77.5)
After first diagnosis metastatic disease	18 (22.5)
Endocrine treatment before metastatic biopsy	
SERM only	32 (40.0)
AI only	18 (22.5)
SERM and AI	24 (30.0)
No endocrine treatment	6 (7.5)
Duration endocrine treatment before metastatic biopsy	
<2 years	21 (26.3)
2 to 4 years	14 (17.5)
>4 years	45 (56.3)

*AI* aromatase inhibitor, *ET* endocrine therapy, *SERM* selective estrogen receptor modulator

^a^The total number is >80 since several metastatic samples were analyzed for some patients

^b^This includes metastatic samples collected up to 3 months after first diagnosis of metastatic disease

### Incidence and distribution of *ESR1* mutations in the retrospective metastatic ILC cohort

We assessed the presence of five different ESR1 mutations: E380Q, Y537S/C/N, and D538G. Five patients (6%) had a unique *ESR1* mutation in the primary tumor (*n* = 3) or axillary lymph node metastasis (*n* = 2), which was not detected in the matched distant metastasis (Table 2). These mutations showed a variant allele fraction ranging between 0.1 and 2.0% and were distributed as follows: D538G (*n* = 3), Y537S (*n* = 1), and Y537N (*n* = 1). The metastases from seven patients (9%) harbored *ESR1* mutations, which were absent from the interrogated primary samples (Table 3). Of these, patient #6 harbored the Y537S mutation in the skin metastasis and the D538G mutation in the axillary lymph node metastasis. Of interest, for two of these patients, two different metastatic samples collected at a different time of disease evolution displayed a different *ESR1* mutational status. The first metastatic biopsy of patient #2 was taken at first diagnosis of recurrence and did not harbor an *ESR1* mutation, while the second, taken at progression two years later after she had received an aromatase inhibitor, presented the D538G mutation. Patient #78, who was diagnosed with de novo metastatic disease, also had two metastases that were analyzed, the first taken at diagnosis which was negative and the second taken 1.3 years later after being treated with letrozole, which harbored the E380Q mutation.

**Table 2:** Characteristics of patients and samples from the retrospective metastatic ILC cohort with mutated primary mammary or positive axillary lymph node samples

	Pt6	Pt38	Pt48	Pt54	Pt103
*ESR1* mutations (AF)	D538G (1%)-both PLN samples	D538G (2%)-in only 1 of the 4 P samples	Y537N (0.1%) in PLN	D538G (2%) in P	E380Q (5.5%) in P
Nr of P samples evaluated	2	4	1	1	1
Nr of PLN samples evaluated	2	0	1	1	0
Nr of M samples evaluated	1	2	1	22	1
Age at P diagnosis	49	65	45	33	55
PgR (P)	+	−	+	+	+
HER2 (P)	−	−	−	−	+
Grade (P)	3	1	2	2	2
Ki67 (P)	10	5	37	15	NA
Histotype (P)	Mixed NC	Classic	Trabecular	Classic	Mixed NC
pT	1	2	2	1	4
pN	1	2	3	3	0
De novo metastatic	Yes	No	No	No	No
Type ET	AI	SERM + AI	SERM	AI	AI
Duration ET (yrs)	7.2	8	5	2.8	1.4
Setting ET	Met	Adj	Adj	Adj	Met
Time between diagnosis and sampling	7.8	0	0	0	0
Organ samples	Bone	Peritoneum	Bone	Liver	Peritoneum

*AF* allelic frequency, *AI* aromatase inhibitor, *ET* endocrine therapy, *NA* not available, *Mixed NC* mixed non-classic, *P* primary, *PLN* positive lymph node, *SERM* selective estrogen receptor modulator

**Table 3:** Characteristics of patients and samples from the retrospective metastatic ILC cohort with mutated metastatic samples

	Pt1	Pt2	Pt6	Pt58	Pt62	Pt67	Pt78
*ESR1* mutations (AF)	Y537S (0.24%)	D538G (22.3%)	Y537S (27%)	D538G (16.9%)	D538G (15.0%)	Y537S (8.0%) Y537N (6.4%) D538G (0.8%)	E380Q (5.5%)
Nr of primary samples (P & PLN) evaluated	7	3	5	2	2	2	1
Age	45	42	49	52	61	54	55
PgR	+	−	+	+	−	+	+
HER2	−	−	−	−	−	−	+
Grade	2	2	3	2	2	NA	2
Ki67	20	10	10	20	18	15	NA
Histotype	Trabe-						
cular	Mixed NC	Mixed NC	Trabecular	Classic	Classic	Mixed NC	
pT	1	1	1	2	2	1	4
pN	1	1	1	2	3	3	0
De novo metastatic	Yes	No	Yes	No	No	No	Yes
Type	AI	SERM + AI	AI	AI	AI	SERM	AI
Duration (yrs)	3.8	2.8 + 1.9	7.2	8	6.8	4.7	1.4
Setting	Met	Adj+					
met	Met	Adj	Adj	Adj	Met		
Time between diagnosis and sampling	3.9	2.0	7.8	0	0	0	1.3
Organ samples	Bone	Bone	Bone	Liver	Bone	Peritoneum	Peritoneum

Patients 6 and 78 had metastases that were sampled at different times during disease evolution and only the second one harboring the ESR1 mutation was reported in this table

*AF* allelic frequency, *AI* aromatase inhibitor, *ET* endocrine therapy, *NA* not available, *Mixed NC* mixed non-classic, *P* primary, *PLN* positive lymph node, *SERM* selective estrogen receptor modulator

### Association of *ESR1* mutations with clinical and pathological features

We explored the associations between the presence of *ESR1* mutations and clinico-pathological features. All but one patients with *ESR1* mutations were ≤ 55 years old at diagnosis and had axillary lymph node metastasi(e)s. Also all but one patients were treated with aromatase inhibitors, in the adjuvant and/or metastatic setting before the metastatic biopsy was taken. This means that the frequency of ESR1-mutated metastases is very different according to endocrine therapy the patients received before their metastatic biopsy: SERM (1/32, 3.1%), AI only (5/18, 27.8%), SERM + AI (1/24, 4.2%), and no endocrine treatment (0/6, 0%). Three of the seven patients were de novo metastatic, however for two of them their biopsy was taken several years after diagnosis. Of interest, the metastasis from patient #67 harbored three different *ESR1* mutations, however this patient received only adjuvant tamoxifen as endocrine treatment. We did not observe any association with the specific histologic ILC variants and none of the metastatic samples that lost ER expression presented an *ESR1* mutation.

### Comparison of the prevalence and distribution of *ESR1* mutations between IDC and ILC patients

**Fig. 1:**
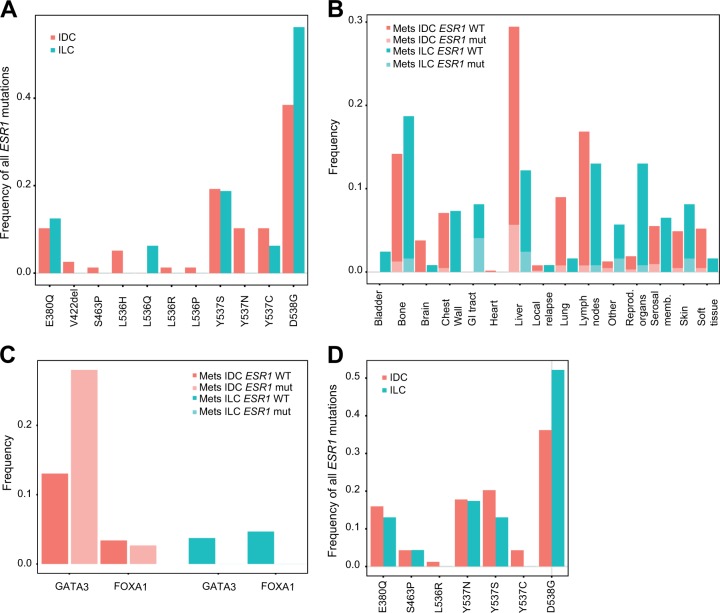
Comparison of prevalence and distribution of *ESR1* mutations in the IDC and ILC metastatic disease. **a** Frequency of the different *ESR1* mutations in IDC and ILC in the metastases from the MSKCC-IMPACT cohort. **b** Distribution of the different types of metastases sequenced from IDC and ILC patients, and proportion of the *ESR1-*mutated (mut) and wild-type (WT) metastases in the MSKCC-IMPACT cohort. **c** Co-occurrence of *ESR1* mutations with *GATA3* and *FOXA1* mutations in IDC and ILC patients in the MSKCC-IMPACT cohort. **d** Frequency of the different *ESR1* mutations in IDC and ILC in ctDNA from the SoFEA and PALOMA-3 trials

To compare the prevalence and distribution of *ESR1* mutations between IDC and ILC patients, we first interrogated the metastases from the MSKCC-IMPACT cohort (Fig. 1a–c). The distribution of the ILC metastases is very similar to what we observed in our retrospective series (Fig. 1b). We observed no statistically significant difference in the prevalence of *ESR1* mutations, with 11.9% (71/595) of the IDC and 13.8% (16/116) of the ILC patients harboring a mutation in at least one of the investigated metastases (*p* = 0.540, Fisher’s exact test). We then compared the types of mutations and observed that the most frequent mutation both in IDC and ILC is the D538G mutation, present in 38.5 and 56.3% of the IDC and ILC mutated samples, respectively. Some of the less commonly reported mutations such as V422del, S463P, L536H/P/R, and Y537N were only observed in metastases from IDC patients. Of note, in the metastases from the ILC patients, the only additionally detected mutation that we found and that was not part of the five mutations we were interrogating in our series was the L536Q mutation present in one metastasis. In this series, we could not assess the presence of the mutations in the matched primary samples, nor the ER status in the primary and metastatic disease or the association with treatment, as these data were not available. Given the key role of FOXA1 and GATA3 in the transcription factor complex of ER,^29^ we aimed at investigating the co-occurrence of mutations present in these genes with *ESR1* mutations. Consistently with what has been observed in the primary disease,^3,5^ we noticed a higher prevalence of *GATA3* mutations in IDC (14.8%) compared to ILC (3.4%) metastases (*p* < 0.001, Fisher’s exact test). The only statistically significant association we observed was between *ESR1* and *GATA3* mutations in IDC metastases, with 28.0% of the *ESR1*-mutated metastases harboring also *GATA3* mutations, compared to only 13.0% of the *ESR1* wild-type metastases (*p* = 0.002, Fisher’s exact test).

We further compared the prevalence and distribution of *ESR1* mutations identified in ctDNA between IDC and ILC patients in the SoFEA (NCT00253422) and PALOMA-3 (NCT01942135) trials (Fig. 1d). In these trials, the *ESR1* mutational status was analyzed in 606 patients (445 from PALOMA-3 and 161 from SoFEA): 416 IDC, 76 ILC, and 117 with other histologies. There was no statistically significant difference in the overall incidence of *ESR1* mutations between the two main histologies with 29.6 and 26.3% reported in samples from IDC and ILC patients, respectively (*p* = 0.680, Fisher’s exact test). Consistently with the above results, we further did not observe any difference in the distribution of the *ESR1* mutations between the two histologies.

## Discussion

In this study, we aimed first at evaluating *ESR1* mutations in a unique retrospective multi-centric cohort of metastatic ILC and second at comparing their prevalence and distribution to metastatic IDC using external cohorts. In our retrospective cohort, *ESR1*-mutated metastases were detected in 9% of the patients. In MSKCC-IMPACT,^18^ we observed a slightly higher rate of 13.8% and 11.9% in metastases from ILC and IDC patients. The lower rate observed in our series might potentially be explained by the fact that only 52% of the patients were treated with aromatase inhibitors before their metastatic biopsy and by the fact that most of the metastatic biopsies (78%) were taken early in the metastatic phase. We could however not compare these two cohorts given the lack of publicly available pathological and clinical data for MSKCC-IMPACT. The even higher rates observed in SoFEA and PALOMA-3^24^ (29.6% and 26.3% in IDC and ILC patients, respectively) are in line with the rates observed in ctDNA studies^15,21–25^ and with the fact that the majority of the patients received prior aromatase inhibitors. Globally, these comparisons revealed no difference in prevalence of *ESR1* mutations between the two histologies. These results are also in line with two previous studies.^27,30^ Bartels et al. reported *ESR1* mutations in 11/% (11/118) and 11.4% (14/113) of IDC and ILC metastases, however this study was limited to bone metastases.^30^ Toy et al. reported *ESR1* mutations in 10.3% (72/698) and 14.2% (20/141) of IDC and ILC samples, respectively, however these were a mixture of primary tumors and metastases from recurring patients.^27^

With regard to the type of mutations present in ILC, we observed that D538G is the most common mutation, present in the metastatic disease from 57.1%, 56.3%, and 60.0% of the ILC patients with *ESR1*-mutated metastases from our series, MSKCC-IMPACT, and the SoFEA/PALOMA-3 ctDNA, respectively. Although these values were numerically higher in ILC compared to IDC patients, the difference was not statistically significant. It has recently been demonstrated that the different activating *ESR1* mutations do promote a metastatic phenotype^31^ but are not similar with regard to the efficacy of ER antagonists.^27^ For instance, Toy et al. observed that Y537S-mutated tumors, as opposed to tumors harboring D538G, E380Q, or S463P mutations, are associated with in vivo resistance to fulvestrant and should therefore be treated with a more potent ER antagonist.^27^ The fraction of Y537S mutations among all *ESR1* mutations was variable across the series: 42% in our series, 18.8% in MSKCC-IMPACT, and 15% in SoFEA/PALOMA-3. This suggests that the type of *ESR1* mutation should be carefully evaluated for optimal endocrine treatment decision.

This study is to the best of our knowledge the largest study of ER-positive BC comparing the presence of *ESR1* mutations in matched primary and metastatic samples. Our data do not support the hypothesis that rare *ESR1*-mutated clones are selected for in the primary tumor during disease progression since we did not detect the mutations identified in the primary disease in the matched metastases. Nevertheless, we cannot rule out that these mutations could be present in another metastatic lesion. Also, we did not detect the mutations present in the metastases in the matched primary samples, although here we cannot exclude that we missed the minor subclone present in the primary tumor despite having interrogated several geographical regions whenever possible with a very sensitive technology.

The main limitations of our study are linked to the retrospective nature of our cohort, with the inherent heterogeneity in treatment and timing of metastatic sampling. Also, the choice of ddPCR was implying that only pre-defined mutations could be investigated. The full spectrum of *ESR1* mutations was however interrogated in the MSKCC-IMPACT cohort.^18^ Only the L536Q mutation was detected in one ILC metastasis from this cohort in addition to the five mutations we were focusing on in our retrospective cohort, suggesting that we did not miss too many mutations in our cohort. In our series, ddPCR was chosen for its high-sensitivity to increase the chance of detecting rare mutations. Indeed a study comparing NGS and ddPCR showed that they could detect three times more *ESR1* mutations with ddPCR than NGS.^32^ Finally, the last limitation of this study was that it focused on *ESR1* mutations only, and was therefore not reporting on other potential mechanisms of endocrine resistance involving directly (*ESR1* gain/amplification/translocation/fusion) or indirectly *ESR1* (such as partners from the transcription factor complex^29^). We nevertheless explored the co-occurrence of mutations present in two key partners, *GATA3* and *FOXA1*, known to be frequently altered in ER-positive BCs. We only observed a positive association between *ESR1* and *GATA3* mutations in metastases from IDC patients, the biological implications of which should be further investigated through functional studies.

To conclude, we have shown here using the largest series of matched primary/metastasis ILC cohort and an ultra-sensitive technology, that there was no patient presenting an identical *ESR1* mutation in the early setting and in metastatic disease. Furthermore, the study did not identify differences in terms of prevalence and type of *ESR1* mutations between the two most common BC histological subtypes.

## Patients and methods

### Patients and samples from the multi-centric retrospective ILC cohort

We considered the patients from six European Institutions (Institut Jules Bordet-Brussels, Cliniques Universitaires Saint-Luc-Brussels, GZA Ziekenhuizen-Antwerp, Institut Paoli-Calmettes-Marseille, Institut Curie-Paris, Istituto Europeo di Oncologia-Milan) that were fulfilling the following criteria: (1) no distinct invasive neoplastic components other than ILC at central revision; (2) ER-positive status of the primary tumor; (3) minimal tumor cellularity of 20% (if < 20%, then only considered if macrodissection could be done); (4) availability of >100 ng of DNA from a formalin-fixed paraffin-embedded (FFPE) block of the primary tumor, metastasis, and non-invaded tissue. Whenever possible, multiple FFPE blocks were considered for the primary tumor, the axillary, and distant metastases. DNA extraction from FFPE samples was performed using the QIAamp DNA FFPE Tissue Kit and concentration was measured using the Qubit fluorometer (Life technologies). The project has been approved by the ethics committee of the Institut Jules Bordet (N°2504). Given the retrospective nature of the study, the ethics committee did not require the patients to sign an informed consent.

### ddPCR

The presence of the mutations E380Q, Y537S/C/N, and D538G was assessed using custom designed mutation-specific assays in a total reaction volume of 23 µl consisting in 1x ddPCR supermix for probes without dUTP, 0.9 µM each primer, 0.25 µM probe, and 10–100 ng of DNA. All ddPCR experiments were performed using the in house Bio-Rad QX200 System. ddPCR assay specificity was assessed using negative and positive controls, consisting of normal DNA from breast reduction and synthetic oligonucleotides harboring the mutations of interest, respectively. QuantaSoft v.1.7.4 software (Bio-Rad) was used for data analysis. Sample positivity was determined following Bio-Rad guidelines. Absolute copies of mutant DNA and wild-type DNA were estimated from the Poisson distribution.

### *ESR1* mutations in external cohorts

We downloaded the targeted sequencing and clinical data from MSKCC-IMPACT^18^ from cbioportal.org^19,20^ in April 2018. We considered only the metastases from female BC patients with the Oncotree code “BRCANOS”, “BRCNOS” and “IDC” code as IDC patients and with the “ILC” code as ILC patients. With regard to the *ESR1* mutations, we only considered those reported to be “Gain-of-function” or “Likely Gain-of-function” according to OncoKB.^33^ For the *GATA3* and *FOXA1* mutations, we considered the mutations annotated as “hotspot” or “3D hotspot” or “Likely Oncogenic” or “Predicted Oncogenic” in cbioportal.org. We further queried the results obtained in ctDNA from the SoFEA (NCT00253422) and PALOMA-3 (NCT01942135) trials.^24^ In these studies, the presence of the *ESR1* mutations was assessed by ddPCR.

### Statistical analyses

The associations between the *ESR1* mutational status and clinico-pathological variables were assessed using the Fisher’s exact test. All statistical tests were two-sided at the 0.05 significance level and conducted under R 3.4.1 (http://www.r-project.org/).

### Reporting summary

Further information on experimental design is available in the Nature Research Reporting Summary linked to this article.

## Supplementary information

Supplementary Table 1

Reporting Summary

## Data Availability

The authors declare that data supporting the findings of this study are available within the paper and its supplementary information files or available online for the MSKCC-IMPACT cohort on cbioportal.org.^19,20^ Data from the SoFEA (NCT00253422) and PALOMA-3 (NCT01942135) trials have been reported previously.^24^

## Supplementary information

**Supplementary information** accompanies the paper on the *npj Breast Cancer* website (10.1038/s41523-019-0104-z).
