# Supplementary material for: ESR1 mutations in metastatic lobular breast cancer patients
Source: NPJ Breast Cancer. 2019 Feb 22;5:9. doi: 10.1038/s41523-019-0104-z (PMC6384916; doi:10.1038/s41523-019-0104-z)
Supplement: Supplementary file 1 — Supplementary Table 1 [file 41523_2019_104_MOESM1_ESM.pdf]

Supplementary material: Desmedt *et al*. "*ESR1* mutations in metastatic lobular breast cancer"

Supplementary table 1: Patient and samples characteristics from the retrospective series.

ID	Age	First recurrence	Death	Subtype	Size	PLN	ER_Prim	ER_Met	PgR_Prim	PgR_Met	HER2_Prim	HER2_Met	Ki67_Prim	Grade_Prim	Site_Met1_Biopsy	Site_Met2_Biopsy	HT_BMB	Tam_BMB	AI_BMB	Tam_and_AI_BMB	CT_BMB	ESR1_Metastasis	ESR1_Primary	ESR1_PLN
1	45	Bone	Yes	Trabecular	0	Yes	Positive	Positive	Positive	Positive	Negative	Negative	20	3	GI tract	NA	Yes	No	Yes	No	No	Y537S (0,24%)	NA	NA
2	42	Bone	Yes	Mixed non-classic	1	Yes	Positive	Positive	Positive	Positive	Negative	Negative	40	3	Bone	Skin	Yes	Yes	Yes	Yes	Yes	D538G (22,3%)	NA	NA
3	64	Lung	Yes	Trabecular	1	No	Positive	Positive	Negative	Negative	Negative	Negative	10	2	Skin	NA	Yes	Yes	Yes	Yes	No	NA	NA	NA
4	60	Liver	Yes	Classic	1	Yes	Positive	Positive	Positive	Negative	Negative	Negative	5	2	Liver	NA	Yes	Yes	Yes	Yes	Yes	NA	NA	NA
5	43	Ovary	Yes	Trabecular	1	Yes	Positive	Positive	Positive	Negative	Negative	NA	10	2	Reproductive organs	NA	Yes	Yes	No	No	Yes	NA	NA	NA
6	49	Bone	Yes	Mixed non-classic	0	Yes	Positive	Positive	Positive	NA	Negative	Negative	10	3	Bone	NA	Yes	No	Yes	No	Yes	Y537S (27%)	NA	D538G (1%)
7	57	Bone	Yes	Classic	1	Yes	Positive	Positive	Positive	NA	Negative	NA	5	1	Bone	NA	Yes	No	Yes	No	Yes	NA	NA	NA
9	56	Skin	Yes	Mixed non-classic	1	No	Positive	Positive	Negative	Positive	Negative	Negative	5	3	Lymph Node	NA	Yes	Yes	Yes	Yes	Yes	NA	NA	NA
10	46	Bone	Yes	Mixed non-classic	1	Yes	Positive	Positive	Positive	Negative	Negative	Negative	20	3	GI tract	NA	Yes	No	Yes	No	Yes	NA	NA	NA
11	44	Liver	No	Trabecular	0	No	Positive	NA	Positive	NA	Positive	NA	20	2	Reproductive organs	NA	No	No	No	No	Yes	NA	NA	NA
12	64	Bone	No	Mixed non-classic	1	No	Positive	Positive	Negative	Positive	Negative	Negative	15	2	Bone	NA	No	No	No	No	No	NA	NA	NA
13	44	Ovary	No	Classic	1	Yes	Positive	Positive	Positive	Positive	Negative	Negative	5	2	Reproductive organs	NA	Yes	Yes	No	No	Yes	NA	NA	NA
14	58	Peritoneum	Yes	Classic	1	Yes	Positive	Positive	Positive	Positive	Negative	Negative	40	1	GI tract	GI tract	Yes	Yes	No	No	Yes	NA	NA	NA
15	53	Uterus	No	Mixed non-classic	1	Yes	Positive	Negative	Positive	Negative	Negative	Negative	5	2	Reproductive organs	NA	Yes	Yes	No	No	Yes	NA	NA	NA
16	74	Stomac	Yes	Classic	1	No	Positive	Positive	Positive	Positive	Negative	Negative	5	2	GI tract	NA	Yes	Yes	Yes	Yes	Yes	NA	NA	NA
19	55	pectoralis d controla	No	Mixed non-classic	1	Yes	Positive	Negative	Positive	Negative	Positive	Positive	15	3	Lymph Node	NA	Yes	No	Yes	No	Yes	NA	NA	NA
20	46	breast (multifocal, sa	No	Classic	0	No	Positive	Positive	Positive	Positive	Negative	Negative	10	2	Skin	NA	Yes	Yes	No	No	No	NA	NA	NA
21	51	Bone	No	Mixed non-classic	1	NA	Positive	Positive	Positive	Positive	Negative	Negative	40	3	Bone	NA	Yes	No	Yes	No	Yes	NA	NA	NA
24	78	Peritoneum	Yes	Solid	1	Yes	Positive	NA	Negative	NA	Negative	NA	5	2	Peritoneum	NA	Yes	Yes	No	No	No	NA	NA	NA
25	51	Liver	Yes	Classic	1	Yes	Positive	Positive	Positive	Positive	Negative	Negative	5	1	Reproductive organs	NA	Yes	Yes	Yes	Yes	Yes	NA	NA	NA
27	43	Bone	No	Mixed non-classic	1	No	Positive	Positive	Positive	Positive	NA	Negative	61	2	Bone	NA	Yes	Yes	No	No	Yes	NA	NA	NA
28	56	Bone	Yes	Classic	0	No	Positive	Positive	Positive	Negative	NA	Negative	NA		1	Local Relapse	NA	Yes	Yes	No	No	No	NA	NA	NA
29	34	Bone	Yes	Classic	1	Yes	Positive	Positive	Positive	Negative	NA	Negative	34	2	Reproductive organs	NA	Yes	Yes	Yes	Yes	Yes	NA	NA	NA
30	56	Skin	Yes	Mixed non-classic	1	No	Positive	Positive	Negative	Positive	Negative	Negative	20	2	Skin	NA	Yes	Yes	No	No	No	NA	NA	NA
31	40	Bone	Yes	Classic	1	Yes	Positive	NA	Positive	NA	Negative	NA	5	2	Reproductive organs	NA	Yes	Yes	Yes	Yes	Yes	NA	NA	NA
32	47	Bone	Yes	Classic	1	Yes	Positive	Negative	NA	Negative	NA	Negative	NA		2	Peritoneum	NA	Yes	Yes	Yes	Yes	Yes	NA	NA	NA
33	65	Liver	No	Mixed non-classic	0	No	Positive	Positive	Positive	Negative	Negative	Negative	5	2	Liver	NA	Yes	Yes	No	No	Yes	NA	NA	NA
34	55	Pleura	Yes	Classic	1	Yes	Positive	Positive	Positive	Negative	NA	Negative	NA		1	Local Relapse	Lung	Yes	Yes	Yes	Yes	Yes	NA	NA	NA
35	67	Skin	No	Classic	0	No	Positive	Positive	Positive	Positive	NA	Negative	18	1	Skin	NA	Yes	Yes	No	No	No	NA	NA	NA
36	70	Bone	Yes	Classic	1	Yes	Positive	Negative	Negative	Negative	Negative	Negative	5	1	Skin	NA	Yes	Yes	Yes	Yes	Yes	NA	NA	NA
38	65	Peritoneum	Yes	Classic	1	Yes	Positive	Positive	Negative	Negative	Negative	Negative	5	1	Peritoneum	NA	Yes	Yes	Yes	Yes	Yes	NA	D538G (2%)	NA
39	60	Skin	Yes	Classic	1	Yes	Positive	Positive	Positive	Positive	Negative	Negative	15	1	Skin	NA	Yes	Yes	Yes	Yes	Yes	NA	NA	NA
41	57	Pleura	Yes	Trabecular	1	No	Positive	Positive	Positive	Negative	Negative	Negative	10	2	Lung	NA	Yes	Yes	No	No	No	NA	NA	NA
42	48	Pleura	Yes	Classic	1	Yes	Positive	Positive	Positive	Negative	Positive	Negative	29	3	Lung	NA	Yes	Yes	No	No	Yes	NA	NA	NA
43	38	Colon	Yes	Classic	1	Yes	Positive	Positive	Positive	Positive	Negative	Negative	19	2	GI tract	NA	Yes	Yes	No	No	Yes	NA	NA	NA
44	63	Bone	No	Trabecular	0	No	Positive	Positive	Positive	Positive	Negative	Negative	21	2	Bone	NA	Yes	Yes	Yes	Yes	No	NA	NA	NA
45	63	Bone	No	Trabecular	0	Yes	Positive	Positive	Positive	Positive	Negative	Negative	27	3	Bone	NA	Yes	Yes	No	No	Yes	NA	NA	NA
46	53	Skin	No	Classic	1	No	Positive	Positive	Positive	Positive	Negative	Negative	18	2	Skin	NA	No	No	No	No	No	NA	NA	NA
48	45	Bone	No	Trabecular	1	Yes	Positive	Positive	Positive	Positive	Negative	Negative	37	2	Bone	NA	Yes	Yes	No	No	Yes	NA	NA	Y537N (0,1%)
50	58	Bone	No	Classic	0	No	Positive	Positive	Positive	Positive	Negative	Negative	18	2	Bone	NA	Yes	Yes	Yes	Yes	No	NA	NA	NA
52	47	Bone	No	Classic	1	Yes	Positive	Positive	Positive	Positive	Positive	Negative	6	2	Bone	NA	Yes	Yes	No	No	Yes	NA	NA	NA
54	33	Liver	Yes	Classic	1	Yes	Positive	Positive	Positive	Positive	Negative	Positive	15	2	Liver	NA	Yes	No	Yes	No	Yes	NA	D538G (2%)	NA
55	51	Bone	No	Classic	1	Yes	Positive	Positive	Positive	Positive	Negative	Negative	6	2	Bone	NA	Yes	Yes	No	No	Yes	NA	NA	NA
56	39	Bone	Yes	Classic	0	Yes	Positive	Positive	Positive	Negative	Negative	Negative	19	2	Bone	NA	Yes	Yes	No	No	Yes	NA	NA	NA
57	51	Bone	No	Trabecular	0	Yes	Positive	Positive	Positive	Negative	Negative	Negative	27	2	Bone	NA	Yes	Yes	Yes	Yes	Yes	NA	NA	NA
58	52	Liver	No	Trabecular	1	Yes	Positive	Positive	Positive	Positive	Negative	Negative	20	2	Liver	NA	Yes	No	Yes	No	Yes	D538G (16,9%)	NA	NA
59	61	Liver	No	Alveolar	1	No	Positive	Positive	Positive	Positive	Negative	Negative	16	2	Bone	NA	Yes	Yes	No	No	No	NA	NA	NA
60	73	Liver	No	Trabecular	1	No	Positive	Positive	Positive	NA	Negative	Negative	25	2	Liver	NA	Yes	Yes	No	No	No	NA	NA	NA
62	61	Bone	No	Classic	1	Yes	Positive	Positive	Negative	Positive	Negative	Negative	18	2	Bone	NA	Yes	No	Yes	No	Yes	D538G (15%)	NA	NA
63	50	Bone	No	Classic	1	Yes	Positive	Positive	Positive	Positive	Negative	Negative	15	2	Bone	NA	Yes	Yes	No	No	No	NA	NA	NA
64	71	Pleura	No	Classic	1	Yes	Positive	Positive	Positive	Negative	Negative	Negative	17	2	Lung	NA	Yes	No	Yes	No	No	NA	NA	NA
65	58	Bone	No	Classic	1	Yes	Positive	Positive	Positive	Negative	Negative	Negative	22	2	Bone	NA	Yes	No	Yes	No	Yes	NA	NA	NA
66	46	Liver	No	Trabecular	1	Yes	Positive	Positive	Positive	Negative	Negative	Negative	23	3	Liver	NA	Yes	Yes	No	No	Yes	NA	NA	NA
67	54	Peritoneum	Yes	Classic	0	Yes	Positive	Positive	Positive	Positive	Negative	Negative	15	2	Peritoneum	NA	Yes	Yes	No	No	Yes	Y537S (8%); Y537N (6,	NA	NA
68	56	Pleura	Yes	Classic	1	No	Positive	Positive	Positive	Negative	Negative	Negative	25	2	Lung	NA	Yes	Yes	No	No	Yes	NA	NA	NA
70	60	Lung	Yes	Trabecular	1	Yes	Positive	Positive	Positive	Positive	Negative	Negative	11	2	Lung	NA	Yes	Yes	No	No	Yes	NA	NA	NA
71	41	Pleura	Yes	Classic	1	No	Positive	Positive	Positive	Negative	Negative	Negative	8	2	Lung	NA	Yes	Yes	No	No	No	NA	NA	NA
72	55	Bone	No	Classic	0	Yes	Positive	NA	Positive	NA	Negative	NA	10	1	Reproductive organs	NA	Yes	Yes	No	No	No	NA	NA	NA
74	60	Cervix	No	Classic	1	Yes	Positive	NA	Positive	NA	Negative	NA	15	2	Lymph Node	NA	Yes	No	Yes	No	Yes	NA	NA	NA
75	69	Skin	Yes	Classic	1	Yes	Positive	NA	Positive	NA	Negative	NA	20	1	Reproductive organs	NA	Yes	Yes	Yes	Yes	Yes	NA	NA	NA
78	55	Pleura	Yes	Mixed non-classic	NA	No	Positive	Positive	Positive	Negative	Negative	NA	NA		2	Lung	Peritoneum	No	No	No	No	No	E380Q (5,5%)	NA	NA
79	65	Bone	No	Classic	1	Yes	Positive	Positive	Positive	Negative	Negative	Negative	NA		2	GI tract	NA	Yes	Yes	Yes	Yes	Yes	NA	NA	NA
80	72	Ovary	Yes	Classic	1	Yes	Positive	Positive	Positive	Negative	Negative	Negative	40	2	Reproductive organs	NA	Yes	Yes	No	No	Yes	NA	NA	NA
81	54	Liver	No	Mixed non-classic	1	Yes	Positive	Negative	Positive	Negative	Positive	Positive	25	1	Skin	NA	Yes	No	Yes	No	Yes	NA	NA	NA
82	69	Skin	Yes	Mixed non-classic	1	Yes	Positive	NA	Positive	NA	Negative	NA	60	3	Skin	NA	Yes	Yes	No	No	No	NA	NA	NA
85	81	Bone	Yes	Mixed non-classic	1	Yes	Positive	Positive	Negative	Negative	Positive	Positive	NA		2	Lymph Node	NA	Yes	No	Yes	No	Yes	NA	NA	NA
87	64	Peritoneum	No	NA	0	No	Positive	Positive	Positive	Positive	Negative	Negative	NA	NA	Peritoneum	NA	No	No	No	No	No	NA	NA	NA
89	58	Uterus	No	Classic	NA	No	Positive	Positive	Positive	Positive	Negative	Negative	NA		1	Reproductive organs	Reproductive organs	No	No	No	No	No	NA	NA	NA
92	50	Liver	No	Solid	1	No	Positive	Positive	Positive	Positive	Negative	Negative	NA		2	Liver	NA	Yes	Yes	No	No	No	NA	NA	NA
100	49	Peritoneum	Yes	Classic	1	Yes	Positive	Positive	Positive	Negative	Negative	Negative	NA		1	Peritoneum	NA	Yes	no	yes	no	yes	NA	NA	NA
101	58	Skin	Yes	Classic	0	No	Positive	Negative	NA	Negative	NA	NA	NA		1	Skin	NA	Yes	no	yes	no	no	NA	NA	NA
102	52	Skin of the thoracic v	Yes	Classic	1	Yes	Positive	Positive	Negative	Negative	Negative	Negative	NA		2	Skin	NA	Yes	yes	yes	yes	no	NA	NA	NA
103	51	Bone	Yes	Classic	1	Yes	Positive	Positive	Negative	Negative	Negative	Negative	NA		2	Bone	NA	Yes	yes	yes	yes	yes	NA	Y537S (0,21%)	NA
104	47	Skin	No	Classic	0	No	Positive	Positive	Positive	Positive	Negative	Negative	NA		2	Skin	NA	Yes	yes	yes	yes	no	NA	NA	NA
105	71	Bone	No	Classic	1	Yes	Positive	NA	Positive	NA	Negative	Negative	NA		3	Bone	NA	Yes	yes	yes	yes	yes	NA	NA	NA
106	75	Liver	Yes	Solid	0	No	Positive	Positive	Positive	Positive	Negative	Negative	13	2	Liver	NA	Yes	yes	no	no	no	NA	NA	NA
107	73	Lymph nodes (supra	Yes	Mixed non-classic	1	Yes	Positive	Positive	Positive	Negative	Negative	Negative	40	3	Lymph Node	NA	Yes	no	yes	no	yes	NA	NA	NA
108	44	Ovary	Yes	Classic	0	Yes	Positive	Positive	Positive	Positive	Negative	Negative	30	3	Reproductive organs	NA	Yes	yes	yes	yes	yes	NA	NA	NA
109	51	LND axilla	Yes	Classic	0	No	Positive	Positive	NA	Negative	Negative	Negative	NA		2	Local Relapse	NA	Yes	yes	yes	yes	no	NA	NA	NA
110	61	Lymph nodes (supra	Yes	Mixed non-classic	1	Yes	Positive	Positive	Positive	Positive	Negative	Negative	NA		3	Lymph Node	NA	Yes	no	yes	no	yes	NA	NA	NA

Description of the columns:

**ID**	
**Age**	Age at Diagnosis
**Menopause_Status**	Post/Pre
**Site_of_first_distant_recurrence**	
**Death**	Yes/No
**Main_Subtype**	Most common subtype
**T_size_binary**	0 = <2cm / 1 = >2cm
**Positive_nodes**	Yes/No
**pN**	
**ER_prim**	Positive/Negative
**ER_met**	
**PgR_prim**	Positive/Negative
**PgR_met**	
**HER2_prim**	Positive/Negative
**HER2_met**	
**Ki67_prim**	
**Grade_prim**	
**Site_Met1_Biopsy**	First met biopsy location
**Tam_BMB**	Received Tamoxifen before Met Biopsy : Yes/No
**AI_BMB**	Received Aromatase Inhibitor before Met Biopsy : Yes/No
**Tam_and_AI_BMB**	Received Tamoxifen and Aromatase Inhibitor before Met Biopsy : Yes/No
**CT_BMB**	Chemotherapy before met biopsy
